# Dimensionality of the Mental Toughness Questionnaire (MTQ48)

**DOI:** 10.3389/fpsyg.2021.654836

**Published:** 2021-07-23

**Authors:** John L. Perry, Doug Strycharczyk, Neil Dagnall, Andrew Denovan, Kostas A. Papageorgiou, Peter J. Clough

**Affiliations:** ^1^Department of Psychology, Mary Immaculate College, Limerick, Ireland; ^2^Department of Psychology, Manchester Metropolitan University, Manchester, United Kingdom; ^3^School of Psychology, Queens University Belfast, Belfast, United Kingdom; ^4^Department of Psychology, University of Huddersfield, Huddersfield, United Kingdom

**Keywords:** mental toughness, MTQ48, exploratory structural equation modeling, psychometrics, bifactor

## Abstract

Currently there is debate as to whether mental toughness is a unidimensional or multidimensional construct. To investigate the dimensionality of the Mental Toughness Questionnaire 48-items (MTQ48), a widely used measure of mental toughness, we examined data from a sample of 78,947 participants. A series of exploratory structural equation models (ESEM) assessed unidimensional, multidimensional, and bifactor solutions. Overall, results supported a bifactor conceptualization of mental toughness. Bifactor analysis was consistent with the use of a general factor score. In conclusion, the authors argue that mental toughness should be considered as an umbrella term representing a general trait comprised of related constructs that provide a psychological advantage in performance and promote positive mental health. Finally, this article identifies limitations in the existing measurement of mental toughness and proposes necessary directions in future research.

## Introduction

Mental toughness (MT) has been conceptualized as the possession of enabling experientially developed and heritable psychological resources (i.e., values, attitudes, emotions, cognitions, and behaviors) that facilitate achievement and promote positive mental health (Coulter et al., 2010; Papageorgiou et al., 2019a,b). More precisely, researchers regard MT as a positive psychological construct, which has important real-world applications. Notably, Clough et al. (2002) delimit MT as the capability to cope with difficulties and to achieve self-defined aims. This conceptualization derives from the notion that MT is a resistance resource that guards against the negative effects of stress across a range of contexts (i.e., sport, education, occupational, and health) (Crust and Keegan, 2010; Lin et al., 2017; Papageorgiou et al., 2019a).

Noting these points, Gucciardi (2017, p. 18) operationalized MT as, “a state-like psychological resource that is purposeful, flexible, and efficient in nature for the enactment and maintenance of goal-directed pursuits.” Collectively, MT delineations reflect the core assumptions that the construct represents an aggregation of personal resources, resides within the individual, is continuous, and helps individuals to deal with everyday hassles and major life events (Gucciardi et al., 2015).

The most widely used instrument to measure MT is the Mental Toughness Questionnaire-48 (MTQ48; Clough et al., 2002; Birch et al., 2017). This developed from Clough’s multidimensional 4Cs model, which posits the existence of discrete, but related factors (i.e., Challenge, Commitment, Control, and Confidence) (Clough and Strycharczyk, 2012). Challenge refers to the extent that individuals perceive barriers and tests as opportunities for self-development. Commitment denotes persistence and the ability to carry out tasks successfully. Control designates the degree to which an individual believes they have influence over their life. Lastly, Confidence represents self-belief in abilities, particularly the capacity to successfully complete tasks.

The 4Cs model draws upon elements of hardiness (Challenge, Commitment, and Control) (Kobasa, 1979). These encourage resilience by motivating coping and social interaction (Maddi and Kobasa, 1984). In recognition of the physical and mental demands of competitive sport, Clough et al. (2002) added Confidence to the hardiness factors. The inclusion of this additional dimension gave Clough’s model a sport-specific (performance-based) focus (Birch et al., 2017).

The Control and Confidence dimensions incorporate nested, subdivided components. In the case of Control, this comprises consideration of emotions and life. For Confidence, this encompasses belief in personal abilities, and capabilities related to dealing with social situations and interactions. As the MTQ48 derives directly from the 4C/6C model, the scale’s performance provides tangible insights into the validity of Clough’s abstraction of MT (see Birch et al., 2017). A review of the relevant literature reveals that psychometric evaluation of the MTQ48 has produced mixed results (Gucciardi, 2018).

Several studies have provided support for the 4C’s across various contexts (Perry et al., 2013, 2015). For example, education (St Clair-Thompson et al. 2014/2017), and health (Gerber et al., 2013). Specifically, Perry et al. (2013) using senior managers, lower and middle managers, clerical/administrative workers, athletes, and students supported the factorial validity of the MTQ48. This sample represented the full domain of possible MT expressions. Following analysis, Perry et al. (2013) concluded that the MTQ48 was a robust psychometric instrument.

An area of concern was the Emotional Control subscale, which demonstrated weak factor loadings and poor internal consistency. Despite this, Perry et al. (2013) advocated cautious retention of the subscale because Emotional Control is an important component of MT. This requires assessing the internal consistency of the emotion subscale prior to analysis. These findings, alongside the results of criterion related validity, indicated that the 4Cs was a valid conceptualization of MT. From the perspective of the MTQ48, the measure requires regular monitoring, item/scale reduction and/or item refinement.

Vaughan et al. (2018) observed acceptable MTQ48 internal consistency at the total and subscale levels using a sample comprising cross elite, amateur, and non-athletes. Evaluation of model fit indicated that the six-factor model possessed acceptable levels of fit, whilst the four-factor model did not fit data well. Within the six-factor model, there were large degrees of misspecification in the factor structures across elite, amateur, and non-athletes. Overall, results cautioned against the use of the measure with elite athletes and suggested that refinement of the MTQ48 at the subscale level was required.

Moreover, other studies have failed to reproduce the 4C solution, and accordingly questioned its appropriateness (Gucciardi et al. 2012/2013). Concerns center on the model’s robustness. Explicitly, fit (i.e., poorly loading items) and applicability to specific samples. Illustratively, confirmatory factor analysis and exploratory structural equation modeling have failed to produce good data fit in athlete and workplace samples (Gucciardi et al., 2012). With misfit evident at local (pattern of factor loadings) and global (model-data congruence) levels. These findings suggest incongruence between the MTQ48 and measurement of the underlying theoretical model. Noting this outcome, Gucciardi et al. (2012) concluded that the MTQ48 might not be a valid measure of MT with student athletes. Based on these observations and further research, Gucciardi et al. (2015) contends that MT is a unidimensional, rather than multidimensional concept.

This notion has proved contentious because other researchers have found evidence for multidimensionality (Perry et al., 2013; Vaughan et al., 2018). Although, they do recommend refinement of the MTQ48 at subscale level. Placing too much emphasis on parsimonious model fit produces a much narrower, unidimensional conceptualization. Whilst this approach has advantages, notably factorial validity, and brevity, it also possesses important disadvantages. Specifically, shorter scales are less sensitive and demonstrate weakened ability to discriminate. This can result in a flatter distribution (Perry et al., 2020). Furthermore, at a practical level the multidimensional approach presents greater potential for applied use, as practitioners can identify individual components of MT to develop. However, in some specific circumstances, an overall, abridged measure can prove useful since it places fewer demands on respondents. This is particularly the case in research that uses a battery of questionnaires.

Using a large sample, this study definitively examined the dimensionality of the MTQ48 and proposed necessary developments. The authors also considered the merits of a bifactor model, whereby MTQ48 items contribute to both subscales and overall representation of MT.

## Methods

### Participants

A sample of 78,947 participant responses were analyzed. Gender was fairly equally split (male = 30,597, 38.76%; female = 28,801, 36.48%; unspecified = 19,549, 24.76%). Age ranged from 11 to 83 (*M* = 35.33, *SD* = 11.12). Data from over 20 nationalities was included. All responses were in English. Occupations ranged from school through to retired individuals. Most of the sample were middle or senior managers, who completed the measure as part of individual coaching.

### Measures

The MTQ48 (Clough et al., 2002) was used to measure mental toughness. This contains 48-items, which require a response on a five-point Likert-type scale anchored from 1 (*strongly disagree*) to 5 (*strongly agree)*.

### Procedure

Participants completed the MTQ48 online between 2014 and 2017. Ethical approval for all data collection was obtained from a departmental ethics committee a United Kingdom higher education institution.

### Data Analysis

Preliminary analyses screened data for missing values and outliers prior to examining distributions. To estimate internal consistency, the authors calculated omega estimates. Main analyses examined a series of structural models using Mplus 8.6 (Muthén and Muthén, 1998-2017). The robust maximum likelihood (MLR) estimator was used throughout. These included unidimensional, multidimensional, and bifactor solutions. Only the six-factor model (not the four-factor) model were explored as part of the multidimensional model, as previous support for this has been unequivocal (e.g., Perry et al., 2013). The unidimensional model includes all 48 items as indicators of an overall MT score.

Within the multidimensional model, the six MTQ factors (Challenge, Commitment, Emotional Control, Life Control, Confidence in Abilities, and Interpersonal Confidence) were postulated as latent variables in an exploratory structural equation model (ESEM; Asparouhov and Muthén, 2009) with target rotation. ESEM models specify that all observed variables (item responses in this case) load onto all latent variables (factors). This is advantageous over traditional methods, such as confirmatory factor analysis with independent cluster models (CFA-ICM), as it does not recognize non-significant cross-loadings as misspecifications (Marsh et al., 2004).

Previous research indicates that ESEM is a more appropriate method for assessing factorial validity in complex models with several factors (Perry et al., 2015). Target rotation designates that “targeted” cross-loadings but not forced (as they would in CFA-ICM) to be zero (Browne, 2001). This method outperforms the default geomin rotation for accuracy in Monte Carlo samples (Myers et al., 2016). As noted by Marsh et al. (2014), the target rotation method is particularly appropriate when ESEM is used in a more confirmatory context, such as when there is a clearly defined *a priori* factor structure.

Analysis next tested a series of increasingly constrained multigroup CFAs evaluated measurement invariance for gender and age. Practitioners often enquire about this information and the large sample provided an opportune moment to test this. This involved four stages. The first assessed configural invariance by replicating the model across groups. The second evaluated metric variance by constraining factor loadings. The third tested scalar invariance by constraining factor loadings and intercepts. Finally, the fourth stage examined residual invariance by constraining factor loadings, item intercepts, and factor means. The assumption of measurement invariance derived from the observation of little or no change (ΔCFI ≤ 0.01; Cheung and Rensvold, 2002) on the increasingly constrained models.

Finally, a bifactor model examined whether it was appropriate to assess simultaneously the composite parts of MT and overall MT. Hence, this solution necessitated the inclusion of a seventh, general factor upon which all items loaded in addition to their loading onto component factors.

Each model fit was assessed via several fit indices. Explicitly, the comparative fit index (CFI) and the Tucker-Lewis index (TLI) functioned as incremental indices and the standardized root-mean-square residual (SRMR) and root-mean square error of approximation (RMSEA) served as absolute fit indices. We cautiously adopted Hu and Bentler’s (1999) recommendations of acceptable fit approaching.95 for incremental fit indices and close to.05 for absolute fit indices. Consistent with the advice of several authors these rules of thumb acted as guidelines rather than definitive criteria (e.g., Marsh et al., 2004; Perry et al., 2015). Regarding parameter estimates, standardized loadings < 0.30 were negligible.

## Results

Preliminary analysis found < 0.1% missing data and no concerning outliers. Owing to the large sample size, normality estimates were very close to zero (skew < 1, kurt < 1). Internal consistency estimates were all satisfactory (ω > 0.70) except for Emotional Control (ω = 0.63). This is consistent with previous literature examining the MTQ48 (e.g., Perry et al., 2013).

The first model tested was the unidimensional model. The model demonstrated poor fit, χ^2^(1,080) = 245513.46, CFI = 0.663, TLI = 0.648, SRMR = 0.058, RMSEA = 0.054 [90% CI = 0.053, 0.054]. However, 43 of the 48 items loaded satisfactorily (>0.30). The high modification indices indicate that it would be quite simple to produce a short unidimensional scale with good fit from this data. Next, analysis tested the multidimensional, six-factor model. The resultant model fit provided reasonable support for this factor structure, χ^2^(855) = 71233.41, CFI = 0.903, TLI = 0.872, SRMR = 0.021, RMSEA = 0.032 [90% CI = 0.032, 0.032]. Examination of factor loadings identified that 30 of the items presented satisfactory loading on their respective scale (Table 1), however, this was not consistent across all factors.

**TABLE 1:** Factor loadings for multidimensional model with target rotation.

Item	Challenge	Commitment	Control		Confidence		*R*^2^
				
			Emotion	Life	Abilities	Interpersonal	
1	**0.12**	0.28	–0.04	–0.16	0.31	0.12	0.37
2	**0.15**	0.08	0.30	0.18	–0.02	0.13	0.28
3	**0.13**	0.06	0.13	0.38	–0.01	0.08	0.27
4	**0.43**	0.14	0.15	0.00	0.18	0.14	0.47
5	**0.49**	0.12	0.10	0.05	–0.04	0.26	0.47
6	**0.09**	0.07	0.06	–0.11	0.21	0.15	0.16
7	**0.25**	0.25	–0.03	–0.06	0.19	0.23	0.41
8	**0.53**	0.17	0.06	0.09	0.05	0.15	0.52
9	–0.03	**0.04**	–0.02	–0.01	0.05	0.00	0.00
10	0.35	**0.25**	0.01	0.05	0.07	0.09	0.32
11	–0.02	**0.39**	0.21	0.19	0.04	0.09	0.41
12	0.47	**0.28**	–0.10	0.16	–0.04	0.06	0.43
13	–0.04	**0.44**	0.16	0.08	0.04	–0.01	0.30
14	0.36	**0.40**	–0.12	0.08	–0.01	0.01	0.37
15	0.10	**0.36**	0.02	0.16	0.06	0.08	0.30
16	–0.12	**0.63**	0.35	–0.08	–0.18	0.05	0.53
17	0.07	**0.50**	0.04	–0.15	0.10	0.10	0.40
18	–0.08	**0.44**	–0.03	0.29	0.14	0.03	0.40
19	–0.02	**0.32**	0.05	0.15	0.08	0.12	0.26
20	0.11	–0.16	**0.39**	0.18	0.15	0.11	0.30
21	0.28	0.09	**0.35**	0.00	–0.05	–0.36	0.24
22	–0.09	–0.11	**0.47**	0.15	0.27	0.13	0.44
23	0.17	0.19	**0.29**	–0.19	0.33	0.00	0.41
24	0.33	–0.01	**0.21**	–0.25	–0.01	–0.29	0.21
25	–0.12	0.62	**0.36**	–0.11	–0.14	0.05	0.54
26	0.01	0.16	**0.20**	–0.13	0.32	0.12	0.32
27	–0.03	0.23	0.00	**−−0.02**	0.40	0.02	0.30
28	–0.07	0.17	–0.13	**−−0.12**	0.26	0.35	0.35
29	0.13	0.25	0.05	**0.35**	–0.04	0.10	0.34
30	0.31	0.02	0.06	**0.16**	0.31	0.08	0.36
31	–0.11	0.21	0.06	**0.42**	0.19	0.02	0.37
32	–0.13	0.21	–0.01	**0.39**	0.15	0.00	0.27
33	0.17	0.16	0.00	**0.38**	0.11	0.16	0.40
34	0.07	0.14	–0.05	0.08	**0.46**	0.06	0.36
35	0.05	0.11	0.07	–0.13	**0.46**	0.19	0.44
36	–0.22	0.08	0.13	0.20	**0.27**	–0.05	0.20
37	0.14	0.02	0.07	0.02	**0.53**	–0.02	0.35
38	0.38	–0.05	0.14	0.13	**0.38**	0.04	0.44
39	0.10	0.10	0.18	0.31	**0.20**	0.14	0.42
40	–0.17	–0.18	0.19	–0.12	**0.39**	0.01	0.20
41	0.06	0.09	0.09	0.47	**0.14**	0.01	0.36
42	–0.12	–0.03	0.45	0.02	**0.25**	0.18	0.41
43	–0.06	–0.04	–0.12	–0.11	0.09	**0.65**	0.41
44	0.09	0.22	–0.19	–0.06	0.20	**0.33**	0.37
45	–0.04	0.08	0.17	0.10	0.12	**0.33**	0.30
46	0.15	0.09	–0.02	–0.02	0.03	**0.45**	0.32
47	0.24	–0.13	0.03	–0.06	–0.14	**0.64**	0.37
48	0.11	0.05	0.05	0.18	–0.16	**0.55**	0.41

**Intended factor loadings highlighted in bold. Item number does not correspond to item order of MTQ48.**

Specifically, the interpersonal Confidence factor performed the best, with all items loading satisfactorily and no items presenting substantive cross-loadings onto other scales. The Commitment scale had eight satisfactory loadings and three < 0.30. Three of the items from this scale loaded also onto the Challenge scale. Slightly over 50% of items presented satisfactory loadings on the Emotional Control, Life Control, and Confidence in Abilities factors and on each of these factors, there were two non-negligible cross-loadings. The Challenge scale performed the worst, as five of the eight loadings were < 0.30.

Given the equivocal findings of previous studies examining the factor structure of the MTQ48, further analysis assessed whether model fit was replicated across random subsamples. Thus, the original sample was randomly divided into 50 subsamples of 1,578 and the same bifactor ESEM with target rotation applied. The results (Supplementary Table 1) presented highly consistent findings for both incremental fit indices (CFI = 0.912–0.951, TLI = 0.878–0.933) and absolute fit indices (SRMR = 0.025–0.020, RMSEA = 0.037–0.025).

Next analysis tested measurement invariance for gender and age using the bifactor model. For age, we defined the following groups: < 21 (*n* = 1,531), 21–30 (*n* = 16,508), 31–40 (*n* = 13,482), 41–50 (*n* = 12,552), > 50 (*n* = 7,101). There were 27,764 (35.20%) with age unspecified. Measurement invariance was supported across gender and age (Table 2) indicating that the obtained outcomes were comprehensive and broadly applicable in research and practice. Further, measurement invariance was tested for gender and supported (Metric ΔCFI < 0.01 = 87.50%; Scalar ΔCFI < 0.01 = 97.50%; Residual ΔCFI = 95.00%) in the 50 subsamples (Supplementary Table 2).

**TABLE 2:** Measurement invariance testing for bifactor model by gender and age.

Model	χ^2^	*df*	Δχ^2^	Δ*df*	CFI	ΔCFI	TLI	SRMR	RMSEA (90% CI)	ΔRMSEA
***Gender* (*n* = 57,480)**										
Configural invariance	45955.82	1626	–	–	0.927	–	0.898	0.019	0.031 (0.031, 0.031)	–
Metric invariance	44258.53	1913	–1697.29	287	0.930	0.003	0.917	0.020	0.028 (0.028, 0.028)	0.003
Scalar invariance	45560.23	1954	1301.70	41	0.928	0.002	0.917	0.021	0.028 (0.028, 0.028)	0.000
Residual invariance	47168.56	1961	1608.33	7	0.925	0.003	0.914	0.025	0.028 (0.028, 0.029)	0.000
***Age* (*n* = 51,174)**										
Configural invariance	45428.99	4065	–	–	0.931	–	0.905	0.019	0.032 (0.031, 0.032)	–
Metric invariance	49285.47	5213	3856.48	1148	0.927	0.004	0.921	0.025	0.029 (0.029, 0.029)	0.003
Scalar invariance	54667.45	5377	5381.98	164	0.918	0.009	0.914	0.027	0.030 (0.030, 0.030)	0.001
Residual invariance	56891.62	5405	2224.17	28	0.914	0.004	0.911	0.036	0.031 (0.030, 0.031)	0.001

The bifactor model produced a stronger model fit than the multidimensional model, χ^2^(813) = 58216.35, CFI = 0.934, TLI = 0.909, SRMR = 0.018, RMSEA = 0.030 [90% CI = 0.030, 0.030]. Factor loadings were significantly larger on the general factor than the subscale factors (Table 3). To further investigate the bifactor model, the authors calculated hierarchical omega and subscale omega to determine the relative variance explained by the general factor and composite subscales, adopting the recommendations of Rodriguez et al. (2016). Explained common variance (ECV) by the general factor (0.72) and its associated hierarchical omega (ω*H* = 0.89) suggest unidimensionality, as does the relative omega value (0.96), which represents the proportion of variance in the multidimensional composite due to the general factor (Dueber, 2017). Explained common variance, hierarchical omega estimates, and relative omega for subscales are presented in Supplementary Table 3. Relative omega for the subscales were substantively different, as Interpersonal Confidence and Emotional Control accounted for greater variance than Commitment.

**TABLE 3:** Factor loadings for bifactor model with target rotation.

Item	MT	Challenge	Commitment	Control		Confidence		*R*^2^
					
				Emotion	Life	Abilities	Interpersonal	
1	0.46	**0.32**	0.10	0.01	–0.04	0.14	0.05	0.37
2	0.46	**−−0.06**	–0.01	0.25	0.13	–0.14	0.05	0.31
3	0.42	**−−0.12**	–0.08	0.06	0.22	–0.13	–0.02	0.28
4	0.65	**0.11**	–0.08	0.11	–0.14	0.01	0.01	0.47
5	0.62	**0.08**	–0.12	0.08	–0.12	–0.20	0.09	0.48
6	0.30	**0.23**	–0.01	0.13	0.03	0.05	0.11	0.18
7	0.57	**0.32**	0.01	0.04	0.01	–0.04	0.11	0.44
8	0.67	**0.11**	–0.13	0.04	–0.11	–0.15	0.00	0.52
9	0.02	0.03	**0.03**	–0.01	0.01	0.03	0.00	0.00
10	0.54	0.06	**0.01**	–0.05	–0.14	–0.04	–0.04	0.33
11	0.55	–0.04	**0.24**	0.10	0.16	–0.03	0.00	0.41
12	0.57	0.00	**−−0.05**	–0.18	–0.16	–0.15	–0.10	0.45
13	0.43	–0.02	**0.30**	0.04	0.05	0.02	–0.07	0.29
14	0.53	0.07	**0.08**	–0.18	–0.15	–0.11	–0.11	0.38
15	0.52	0.05	**0.14**	–0.04	0.10	–0.05	–0.02	0.30
16	0.44	–0.15	**0.59**	0.12	–0.12	–0.05	0.01	0.57
17	0.49	0.25	**0.30**	0.03	–0.04	0.00	0.03	0.40
18	0.53	0.04	**0.22**	–0.09	0.26	0.02	–0.05	0.41
19	0.46	0.06	**0.17**	0.00	0.16	–0.02	0.03	0.27
20	0.39	–0.28	–0.11	**0.22**	–0.03	0.16	0.03	0.32
21	0.12	0.00	–0.01	**0.32**	–0.08	–0.12	–0.31	0.25
22	0.40	–0.16	–0.02	**0.36**	0.16	0.21	0.09	0.44
23	0.48	0.31	0.06	**0.36**	–0.01	0.12	–0.02	0.47
24	–0.05	0.20	–0.08	**0.30**	–0.19	–0.11	–0.22	0.24
25	0.45	–0.15	0.60	**0.13**	–0.16	–0.01	0.01	0.60
26	0.41	0.23	0.09	**0.23**	0.05	0.17	0.08	0.35
27	0.41	0.09	0.13	–0.06	**−−0.03**	0.34	–0.04	0.32
28	0.36	0.22	0.10	–0.11	**0.02**	0.18	0.25	0.35
29	0.53	–0.08	0.04	–0.03	**0.19**	–0.15	–0.02	0.35
30	0.57	–0.08	–0.15	–0.06	**−−0.12**	0.21	–0.06	0.39
31	0.47	–0.05	0.07	0.00	**0.38**	0.05	–0.06	0.39
32	0.37	–0.03	0.07	–0.04	**0.37**	0.02	–0.06	0.29
33	0.60	–0.11	–0.05	–0.10	**0.14**	0.00	0.01	0.40
34	0.50	0.02	0.01	–0.14	–0.05	**0.39**	–0.05	0.40
35	0.49	0.13	0.04	0.01	–0.11	**0.38**	0.10	0.46
36	0.21	–0.04	0.09	0.09	0.26	**0.20**	–0.05	0.20
37	0.45	0.10	–0.09	0.05	–0.05	**0.36**	–0.07	0.35
38	0.59	–0.06	–0.21	0.04	–0.16	**0.25**	–0.08	0.47
39	0.60	–0.18	–0.01	0.03	0.10	**0.13**	0.01	0.42
40	0.02	0.01	–0.02	0.18	0.00	**0.38**	0.05	0.20
41	0.48	–0.17	–0.06	–0.01	0.26	**0.02**	–0.08	0.36
42	0.38	–0.15	0.09	0.30	0.04	**0.26**	0.13	0.41
43	0.32	0.08	0.00	–0.13	–0.03	0.10	**0.49**	0.41
44	0.45	0.30	0.04	–0.11	0.06	0.03	**0.21**	0.38
45	0.46	–0.13	0.08	0.03	0.03	0.13	**0.20**	0.31
46	0.47	0.11	–0.02	–0.03	–0.01	–0.05	**0.29**	0.32
47	0.39	–0.05	–0.13	–0.03	–0.16	–0.12	**0.44**	0.37
48	0.51	–0.12	–0.02	–0.05	0.06	–0.16	**0.35**	0.40

**Intended factor loadings highlighted in bold. Item number does not correspond to item order of MTQ48.**

## Discussion

Building on preceding work, this study further examined the dimensionality of the MTQ48. This was necessary because previous research has produced inconsistent solutions. The current findings supported a bifactor model, indicating the presence of both multi-dimensionality and an underlying general factor. This factor, however, did not account for more variance than the discrete 6Cs. These outcomes advise that the MTQ48 provides valid measurements at both multi and unidimensional levels. Specifically, in applied settings, where factor scores provide nuanced suggestions for personal development, and in academic research using overall scores.

Commensurate with these outcomes, subsequent scale development should continue to assess the relative contribution of subscale scores to global mental toughness (MT). This approach is consistent with the need to regular assess scale dimensionality and refine subscale items (e.g., Emotional Control scale). Indeed, this work is ongoing and will be reported shortly. Ultimately, the authors encourage researchers to focus on producing an improved measure of MT, rather than merely focusing on further investigation of MTQ48 factor structure. This advocacy reflects the fact that psychometric tests need systematic review as understanding of psychological concepts evolves and advances.

A limitation of the present study is that the MTQ48 was designed to assess MT as a multidimensional construct. It is therefore not possible to make claims about MT as operationalized differently by alternative psychometric measures. While the scale and breadth of the sample is a potential strength of the current study, a second limitation is the broad range of individuals within the sample. It is, however, anticipated that by utilizing a very large sample, containing a range of demographic representation, more robust and practically useful models were produced.

In conclusion, the results presented here provide general support for the factorial validity of the MTQ48, particularly with regards to a bifactor model, but highlights specific needs for modification. Firstly, a small number of items require replacing. Secondly, if the scale is to function as a multidimensional scale in practice, a greater distinction between the constructs is required, particularly where ECV is low (i.e., Commitment). Moreover, the recent increase in interest in mental toughness in a broad range of domains, including education, health, sport and business, emphasizes the importance of establishing robust measures and models of mental toughness in light of its increasing prominence.

## Data Availability Statement

The raw data supporting the conclusions of this article will be made available by the authors, without undue reservation.

## Ethics Statement

The studies involving human participants were reviewed and approved by the Manchester Metropolitan University. The patients/participants provided their written informed consent to participate in this study.

## Author Contributions

JP, PC, and DS designed the study. PC and JP were main authors. JP conducted all analyses. DS organized data collection. ND, AD, and KP advised on manuscript contents and contributed to writing and reviewing of drafts. All authors contributed to the article and approved the submitted version.

## Conflict of Interest

DS was Managing Director of AQR International, who distribute the MTQ48. The remaining authors declare that the research was conducted in the absence of any commercial or financial relationships that could be construed as a potential conflict of interest.

## Publisher’s Note

All claims expressed in this article are solely those of the authors and do not necessarily represent those of their affiliated organizations, or those of the publisher, the editors and the reviewers. Any product that may be evaluated in this article, or claim that may be made by its manufacturer, is not guaranteed or endorsed by the publisher.
