# Supplementary material for: Dimensionality of the Mental Toughness Questionnaire (MTQ48)
Source: Front Psychol. 2021 Jul 23;12:654836. doi: 10.3389/fpsyg.2021.654836 (PMC8342756; doi:10.3389/fpsyg.2021.654836)
Supplement: Supplementary file 1 [file Data_Sheet_1.pdf]

Table 1.

Bifactor model fit indices for randomized subsamples of n = 1,578

Subsample (n = 1578)	χ^2 (df = 813)	CFI	TLI	SRMR	RMSEA (90% CI)
1	1998.95	.950	.931	.021	.030 (.029, .032)
2	1720.20	.947	.926	.021	.027 (.025, .028)
3	2309.62	.916	.884	.024	.034 (.033, .036)
4	2067.18	.935	.909	.022	.031 (.030, .033)
5	2236.33	.930	.903	.023	.033 (.032, .035)
6	2307.86	.926	.897	.023	.034 (.033, .036)
7	2256.61	.929	.901	.023	.034 (.032, .035)
8	2397.66	.921	.890	.023	.035 (.034, .037)
9	2278.36	.937	.912	.022	.034 (.032, .035)
10	2323.35	.924	.895	.024	.034 (.033, .036)
11	2236.87	.929	.901	.023	.033 (.032, .035)
12	2095.50	.933	.907	.022	.032 (.030, .033)
13	2069.79	.925	.895	.023	.031 (.030, .033)
14	1993.65	.926	.897	.024	.030 (.029, .030)
15	2389.26	.916	.883	.025	.035 (.033, .037)
16	2630.25	.935	.939	.022	.038 (.036, .039)
17	1961.12	.948	.928	.021	.030 (.028, .032)
18	2425.50	.926	.897	.023	.035 (.034, .037)
19	1961.66	.937	.912	.022	.030 (.028, .032)
20	2089.56	.934	.909	.023	.032 (.030, .033)
21	2646.03	.933	.907	.023	.038 (.036, .039)
22	2191.46	.940	.916	.021	.033 (.031, .034)
23	2228.75	.918	.887	.025	.033 (.032, .035)
24	1868.45	.928	.900	.023	.029 (.027, .030)
25	1772.33	.949	.930	.021	.027 (.026, .029)
26	2587.41	.912	.878	.025	.037 (.036, .039)
27	2291.70	.930	.903	.023	.034 (.032, .036)
28	1709.65	.943	.921	.022	.026 (.025, .028)
29	1821.84	.933	.907	.023	.028 (.026, .030)
30	1989.42	.935	.909	.022	.030 (.029, .032)
31	2297.12	.923	.893	.023	.034 (.032, .036)
32	2087.85	.936	.912	.022	.032 (.030, .033)
33	1965.09	.932	.905	.023	.030 (.028, .032)
34	2044.28	.928	.900	.023	.031 (.029, .033)
35	1900.95	.934	.908	.023	.029 (.027, .031)
36	1829.05	.927	.899	.023	.028 (.026, .030)
37	1614.07	.946	.926	.021	.025 (.023, .027)
38	2260.07	.933	.908	.023	.034 (.032, .035)
39	2079.62	.929	.902	.023	.031 (.030, .033)
40	2059.68	.951	.933	.020	.031 (.030, .033)
41	2089.21	.942	.919	.022	.032 (.030, .033)
42	2176.49	.918	.886	.023	.033 (.031, .034)
43	2583.56	.931	.904	.022	.037 (.036, .039)
44	2131.20	.930	.904	.023	.032 (.030, .034)
45	1812.97	.941	.918	.022	.028 (.026, .030)

46	2104.59	.926	.897	.023	.032 (.030, .033)
47	2067.34	.937	.912	.022	.031 (.030, .033)
48	2216.54	.936	.912	.022	.033 (.031, .035)
49	2042.09	.946	.925	.021	.031 (.029, .033)
50	2209.07	.931	.905	.022	.033 (.031, .034)

Table 2.

Measurement invariance testing for bifactor model by gender and age for randomized subsamples.

Subsample	Model	χ^2	*df*	$\Delta\ \chi^2$	Δdf	CFI	ΔCFI	TLI	SRMR	RMSEA (90% CI)	ΔRMSEA
1 (*n* = 1418)	Configural invariance	2966.19	1626	-	-	.935	-	.910	.026	.034 (.032, .036)	-
	Metric invariance	3563.34	1913	597.15	287	.920	.015	.906	.045	.035 (.033, .037)	.001
	Scalar invariance	3656.83	1954	93.49	41	.918	.002	.905	.043	.035 (.033, .037)	.000
	Residual invariance	4053.96	1961	397.13	7	.899	.019	.884	.069	.039 (.037, .040)	.004
2 (*n* = 1199)	Configural invariance	3121.36	1626	-	-	.916	-	.883	.025	.034 (.032, .036)	-
	Metric invariance	2989.64	1913	-131.72	287	.939	.023	.928	.033	.027 (.025, .029)	.007
	Scalar invariance	3073.69	1954	84.05	41	.937	.002	.927	.034	.027 (.025, .029)	.000
	Residual invariance	3107.96	1961	34.27	7	.935	.002	.926	.035	.027 (.026, .029)	.000
3 (*n* = 1369)	Configural invariance	3103.84	1626	-	-	.910	-	.875	.028	.036 (.034, .038)	-
	Metric invariance	3356.54	1913	252.7	287	.912	.002	.896	.042	.033 (.031, .035)	.003
	Scalar invariance	3486.75	1954	130.21	41	.906	.006	.892	.044	.034 (.032, .036)	.001
	Residual invariance	3612.25	1961	125.5	7	.899	.007	.884	.048	.035 (.033, .037)	.001
4 (*n* = 1150)	Configural invariance	3123.30	1626	-	-	.881	-	.835	.030	.040 (.038, .042)	-
	Metric invariance	3095.71	1913	-27.59	287	.906	.025	.889	.040	.033 (.031, .035)	.007
	Scalar invariance	3167.42	1954	71.71	41	.904	.002	.889	.041	.035 (.033, .035)	.002
	Residual invariance	3241.57	1961	74.15	7	.898	.006	.883	.046	.034 (.032, .036)	.001
5 (*n* = 1440)	Configural invariance	3206.56	1626	-	-	.913	-	.879	.027	.037 (.035, .039)	-
	Metric invariance	3458.31	1913	251.75	287	.915	.002	.900	.036	.033 (.032, .035)	.004
	Scalar invariance	3550.67	1954	92.36	41	.912	.003	.898	.037	.034 (.032, .035)	.001
	Residual invariance	3546.12	1961	-4.55	7	.913	.001	.900	.037	.034 (.032, .035)	.000
6 (*n* = 1346)	Configural invariance	3018.38	1626	-	-	.915	-	.882	.028	.036 (.034, .038)	-
	Metric invariance	3325.05	1913	306.67	287	.913	.002	.898	.037	.033 (.031, .035)	.003
	Scalar invariance	3369.31	1954	44.26	41	.913	.000	.900	.037	.033 (.031, .035)	.000
	Residual invariance	3441.70	1961	72.39	7	.909	.004	.896	.040	.033 (.032, .035)	.000

Subsample	Model	χ^2	*df*	$\Delta\chi^2$	Δdf	CFI	ΔCFI	TLI	SRMR	RMSEA (90% CI)	ΔRMSEA
7 (*n* = 1308)	Configural invariance	2962.79	1626	-	-	.914	-	.880	.028	.035 (.033, .037)	-
	Metric invariance	3271.93	1913	309.14	287	.912	.002	.897	.039	.033 (.031, .035)	.002
	Scalar invariance	3401.47	1954	129.54	41	.907	.005	.892	.041	.034 (.032, .036)	.001
	Residual invariance	3482.37	1961	80.9	7	.902	.005	.887	.043	.034 (.033, .036)	.001
8 (*n* = 1366)	Configural invariance	2983.46	1626	-	-	.920	-	.889	.027	.035 (.033, .037)	-
	Metric invariance	3241.69	1913	258.23	287	.922	.002	.908	.034	.032 (.030, .034)	.003
	Scalar invariance	3309.19	1954	67.5	41	.920	.002	.908	.035	.032 (.030, .034)	.000
	Residual invariance	3350.22	1961	41.03	7	.918	.002	.906	.036	.032 (.030, .034)	.000
9 (*n* = 1386)	Configural invariance	3090.34	1626	-	-	.919	-	.888	.027	.036 (.034, .038)	-
	Metric invariance	3557.25	1913	466.91	287	.909	.010	.893	.037	.035 (.033, .037)	.001
	Scalar invariance	3539.59	1954	-17.66	41	.913	.004	.899	.038	.034 (.032, .036)	.001
	Residual invariance	3632.08	1961	92.49	7	.908	.005	.894	.039	.035 (.033, .037)	.001
10 (*n* = 1482)	Configural invariance	3371.40	1626	-	-	.905	-	.869	.028	.038 (.036, .040)	-
	Metric invariance	3457.13	1913	85.73	287	.916	.011	.901	.037	.033 (.031, .035)	.005
	Scalar invariance	3527.18	1954	70.05	41	.915	.001	.901	.037	.033 (.031, .035)	.000
	Residual invariance	3609.28	1961	82.1	7	.911	.004	.897	.042	.034 (.032, .035)	.001
11 (*n* = 1373)	Configural invariance	3264.79	1626	-	-	.909	-	.873	.028	.038 (.036, .040)	-
	Metric invariance	3407.97	1913	143.18	287	.917	.008	.902	.035	.034 (.032, .036)	.004
	Scalar invariance	3480.14	1954	72.17	41	.915	.002	.902	.036	.034 (.032, .036)	.000
	Residual invariance	3516.23	1961	36.09	7	.913	.002	.900	.039	.034 (.032, .036)	.000
12 (*n* =1437)	Configural invariance	3072.35	1626	-	-	.921	-	.890	.026	.035 (.033, .037)	-
	Metric invariance	3218.59	1913	146.24	287	.928	.007	.915	.034	.031 (.029, .033)	.004
	Scalar invariance	3307.50	1954	88.91	41	.926	.002	.914	.034	.031 (.029, .033)	.000
	Residual invariance	3349.26	1961	41.76	7	.924	.002	.912	.036	.031 (.030, .033)	.000
13 (*n* = 1481)	Configural invariance	2863.71	1626	-	-	.921	-	.890	.027	.032 (.030, .034)	-
	Metric invariance	3069.65	1913	205.94	287	.926	.005	.913	.033	.029 (.027, .030)	.003
	Scalar invariance	3147.20	1954	77.55	41	.924	.002	.912	.034	.029 (.027, .031)	.000
	Residual invariance	3163.85	1961	16.65	7	.923	.001	.911	.035	.029 (.027, .031)	.000

Subsample	Model	χ^2	*df*	$\Delta\chi^2$	Δ*df*	CFI	ΔCFI	TLI	SRMR	RMSEA (90% CI)	ΔRMSEA
14 (*n* = 1497)	Configural invariance	3037.97	1626	-	-	.906	-	.870	.028	.034 (.032, .036)	-
	Metric invariance	3308.03	1913	270.06	287	.907	.001	.891	.037	.031 (.029, .033)	.003
	Scalar invariance	3381.56	1954	73.53	41	.905	.002	.890	.038	.031 (.029, .033)	.000
	Residual invariance	3388.54	1961	6.98	7	.905	.000	.891	.038	.031 (.029, .033)	.000
15 (*n* =1444)	Configural invariance	3208.70	1626	-	-	.903	-	.865	.029	.037 (.035, .039)	-
	Metric invariance	3481.00	1913	272.3	287	.904	.001	.887	.037	.034 (.032, .035)	.003
	Scalar invariance	3569.37	1954	88.37	41	.901	.003	.886	.038	.034 (.032, .036)	.000
	Residual invariance	3585.96	1961	16.59	7	.900	.001	.885	.039	.034 (.032, .036)	.000
16 (*n* = 1375)	Configural invariance	3274.36	1626	-	-	.923	-	.893	.027	.038 (.036, .040)	-
	Metric invariance	3682.10	1913	407.74	287	.917	.006	.903	.038	.037 (.035, .038)	.001
	Scalar invariance	3797.32	1954	115.22	41	.914	.003	.901	.040	.037 (.035, .039)	.000
	Residual invariance	3871.13	1961	73.81	7	.911	.003	.897	.049	.038 (.036, .039)	.001
17 (*n* = 1247)	Configural invariance	2984.46	1626	-	-	.919	-	.888	.027	.037 (.035, .039)	-
	Metric invariance	3167.71	1913	183.25	287	.925	.006	.912	.038	.032 (.030, .034)	.005
	Scalar invariance	3215.29	1954	47.58	41	.925	.000	.913	.039	.032 (.030, .034)	.000
	Residual invariance	3257.36	1961	42.07	7	.923	.002	.911	.040	.033 (.031, .035)	.001
18 (*n* = 1403)	Configural invariance	3342.99	1626	-	-	.902	-	.864	.029	.039 (.037, .041)	-
	Metric invariance	3592.95	1913	249.96	287	.904	.002	.887	.038	.035 (.034, .037)	.004
	Scalar invariance	3673.07	1954	80.12	41	.902	.002	.887	.038	.035 (.034, .037)	.000
	Residual invariance	3756.85	1961	83.78	7	.897	.005	.882	.041	.036 (.034, .038)	.001
19 (*n* = 1402)	Configural invariance	2873.84	1626	-	-	.925	-	.895	.027	.033 (.031, .035)	-
	Metric invariance	3130.29	1913	256.45	287	.926	.001	.913	.036	.030 (.028, .032)	.003
	Scalar invariance	3187.43	1954	57.14	41	.925	.001	.914	.036	.030 (.028, .032)	.000
	Residual invariance	3230.26	1961	42.83	7	.923	.002	.912	.041	.030 (.029, .032)	.000
20 (*n* = 1314)	Configural invariance	2864.83	1626	-	-	.926	-	.898	.027	.034 (.032, .036)	-
	Metric invariance	3054.78	1913	189.95	287	.932	.006	.920	.036	.030 (.028, .032)	.004
	Scalar invariance	3175.97	1954	121.19	41	.927	.005	.916	.037	.031 (.029, .033)	.001
	Residual invariance	3220.27	1961	44.3	7	.925	.002	.914	.040	.031 (.029, .033)	.000

Subsample	Model	χ^2	*df*	$\Delta\chi^2$	Δ*df*	CFI	ΔCFI	TLI	SRMR	RMSEA (90% CI)	ΔRMSEA
21 (*n* = 1213)	Configural invariance	3182.94	1626	-	-	.899	-	.860	.029	.040 (.038, .042)	-
	Metric invariance	3449.32	1913	266.38	287	.901	.002	.883	.040	.036 (.034, .038)	.004
	Scalar invariance	3645.33	1954	196.01	41	.891	.009	.874	.042	.038 (.036, .040)	.002
	Residual invariance	3710.96	1961	65.63	7	.887	.004	.870	.047	.038 (.036, .040)	.002
22 (*n* = 1342)	Configural invariance	3230.05	1626	-	-	.920	-	.889	.026	.037 (.035, .039)	-
	Metric invariance	3416.85	1913	186.8	287	.925	.005	.912	.038	.033 (.031, .035)	.004
	Scalar invariance	3757.55	1954	340.7	41	.910	.015	.896	.038	.036 (.034, .038)	.003
	Residual invariance	3593.81	1961	-163.74	7	.919	.009	.906	.040	.034 (.032, .036)	.002
23 (*n* = 1435)	Configural invariance						Inadmissible				
24 (*n* = 1362)	Configural invariance	2905.57	1626	-	-	.903	-	.865	.028	.034 (.032, .036)	-
	Metric invariance	3032.11	1913	126.54	287	.915	.012	.900	.035	.029 (.027, .031)	.005
	Scalar invariance						Inadmissible				
25 (*n* = 1361)	Configural invariance	2635.75	1626	-	-	.938	-	.914	.026	.030 (.028, .032)	-
	Metric invariance	2892.58	1913	256.83	287	.940	.002	.929	.036	.027 (.025, .029)	.003
	Scalar invariance	2987.54	1954	94.96	41	.937	.003	.927	.037	.028 (.026, .030)	.001
	Residual invariance	3061.48	1961	73.94	7	.932	.005	.922	.045	.029 (.027, .031)	.001
26 (*n* = 717)	Configural invariance	3176.06	1626	-	-	.864	-	.811	.034	.052 (.049, .054)	-
	Metric invariance	3435.70	1913	259.64	287	.866	.002	.842	.049	.047 (.045, .050)	.005
	Scalar invariance	3623.53	1954	187.83	41	.853	.016	.830	.049	.049 (.046, .051)	.002
	Residual invariance	3628.90	1961	5.37	7	.853	.000	.831	.051	.049 (.046, .051)	.000
27 (*n* = 1322)	Configural invariance	3069.45	1626	-	-	.913	-	.880	.028	.037 (.035, .039)	-
	Metric invariance	3364.28	1913	294.83	287	.913	.000	.897	.037	.034 (.032, .036)	.003
	Scalar invariance	3498.44	1954	134.16	41	.907	.006	.893	.039	.035 (.033, .036)	.001
	Residual invariance	3528.28	1961	29.84	7	.906	.001	.892	.040	.035 (.033, .037)	.000
28 (*n* = 1462)	Configural invariance	2783.66	1626	-	-	.924	-	.895	.026	.031 (.029, .033)	-
	Metric invariance	2980.13	1913	196.47	287	.930	.006	.918	.034	.028 (.026, .030)	.003
	Scalar invariance	3051.69	1954	71.56	41	.928	.002	.917	.034	.028 (.026, .030)	.000
	Residual invariance	3130.33	1961	78.64	7	.923	.005	.912	.036	.029 (.027, .030)	.001

Subsample	Model	χ^2	*df*	$\Delta\chi^2$	Δdf	CFI	ΔCFI	TLI	SRMR	RMSEA (90% CI)	ΔRMSEA
29 (*n* = 1450)	Configural invariance	2601.65	1626	-	-	.929	-	.901	.027	.029 (.027, .031)	-
	Metric invariance	3087.43	1913	485.78	287	.914	.015	.899	.037	.029 (.027, .031)	.000
	Scalar invariance	3138.13	1954	50.7	41	.914	.000	.900	.038	.029 (.027, .031)	.000
	Residual invariance	3431.07	1961	292.94	7	.893	.021	.877	.045	.032 (.030, .034)	.003
30 (*n* = 1356)	Configural invariance	3072.88	1626	-	-	.898	-	.859	.029	.036 (.034, .038)	-
	Metric invariance	3278.05	1913	205.17	287	.904	.006	.887	.037	.032 (.031, .034)	.004
	Scalar invariance	3371.95	1954	93.9	41	.900	.004	.885	.038	.033 (.031, .035)	.001
	Residual invariance	3403.38	1961	31.43	7	.898	.002	.883	.039	.033 (.031, .035)	.000
31 (*n* = 1338)	Configural invariance	3163.71	1626	-	-	.898	-	.858	.029	.038 (.036, .040)	-
	Metric invariance	3339.38	1913	175.67	287	.905	.007	.888	.037	.033 (.031, .035)	.005
	Scalar invariance	3394.41	1954	55.03	41	.904	.001	.890	.038	.033 (.031, .035)	.000
	Residual invariance	3474.28	1961	79.87	7	.899	.005	.884	.039	.034 (.032, .036)	.001
32 (*n* = 1115)	Configural invariance	2965.78	1626	-	-	.895	-	.855	.030	.038 (.036, .041)	-
	Metric invariance	3168.57	1913	202.79	287	.902	.007	.884	.039	.034 (.032, .036)	.004
	Scalar invariance	3168.68	1954	0.11	41	.905	.003	.890	.039	.033 (.031, .035)	.001
	Residual invariance	3257.18	1961	88.5	7	.899	.006	.884	.042	.034 (.032, .037)	.001
33 (*n* = 1399)	Configural invariance	2893.30	1626	-	-	.913	-	.879	.028	.033 (.031, .035)	-
	Metric invariance	3199.21	1913	305.91	287	.912	.001	.896	.038	.031 (.029, .033)	.002
	Scalar invariance	3279.83	1954	80.62	41	.909	.003	.895	.038	.031 (.029, .033)	.000
	Residual invariance	3355.35	1961	75.52	7	.904	.005	.890	.041	.032 (.030, .034)	.001
34 (*n* = 1356)	Configural invariance	3108.26	1626	-	-	.903	-	.866	.029	.037 (.035, .039)	-
	Metric invariance	3231.38	1913	123.12	287	.914	.011	.899	.036	.032 (.030, .034)	.005
	Scalar invariance	3285.89	1954	54.51	41	.913	.001	.900	.036	.032 (.030, .034)	.000
	Residual invariance	3300.92	1961	15.03	7	.913	.000	.899	.037	.032 (.030, .034)	.000
35 (*n* = 1421)	Configural invariance	2729.14	1626	-	-	.927	-	.898	.027	.031 (.029, .033)	-
	Metric invariance	2959.70	1913	230.56	287	.931	.004	.918	.034	.028 (.026, .030)	.003
	Scalar invariance	3012.48	1954	52.78	41	.930	.001	.919	.035	.028 (.026, .030)	.000
	Residual invariance	3028.59	1961	16.11	7	.929	.001	.919	.035	.028 (.026, .030)	.000

Subsample	Model	χ^2	*df*	$\Delta \chi^2$	Δ*df*	CFI	ΔCFI	TLI	SRMR	RMSEA (90% CI)	ΔRMSEA
36 (*n* = 1094)	Configural invariance	2602.92	1626	-	-	.909	-	.874	.030	.033 (.031, .035)	-
	Metric invariance	2818.54	1913	215.62	287	.916	.007	.901	.037	.029 (.027, .032)	.004
	Scalar invariance	2868.59	1954	50.05	41	.915	.001	.902	.038	.029 (.027, .032)	.000
	Residual invariance	2915.47	1961	46.88	7	.911	.004	.898	.039	.030 (.028, .032)	.001
37 (*n* = 1379)	Configural invariance	2482.42	1626	-	-	.936	-	.911	.026	.028 (.025, .030)	-
	Metric invariance	2669.14	1913	186.72	287	.943	.007	.933	.034	.024 (.022, .026)	.004
	Scalar invariance	2705.40	1954	36.26	41	.944	.001	.934	.034	.024 (.021, .026)	.000
	Residual invariance	2732.49	1961	27.09	7	.942	.002	.934	.035	.024 (.022, .026)	.000
38 (*n* = 747)	Configural invariance	2663.78	1626	-	-	.914	-	.881	.031	.041 (.039, .044)	-
	Metric invariance	2847.49	1913	183.71	287	.923	.009	.909	.040	.036 (.033, .039)	.005
	Scalar invariance	2909.49	1954	62	41	.921	.002	.909	.041	.036 (.033, .039)	.000
	Residual invariance	2911.81	1961	2.32	7	.921	.000	.909	.041	.036 (.033, .039)	.000
39 (*n* = 788)	Configural invariance	2458.13	1626	-	-	.902	-	.864	.033	.036 (.033, .039)	-
	Metric invariance	2665.30	1913	207.17	287	.911	.009	.895	.043	.032 (.029, .034)	.004
	Scalar invariance	2720.21	1954	54.91	41	.910	.001	.896	.044	.032 (.029, .034)	.000
	Residual invariance	2741.48	1961	21.27	7	.908	.002	.894	.045	.032 (.029, .034)	.000
40 (*n* = 866)	Configural invariance	2802.91	1626	-	-	.925	-	.896	.026	.041 (.038, .043)	-
	Metric invariance	2937.42	1913	134.51	287	.935	.010	.923	.037	.035 (.033, .038)	.006
	Scalar invariance	2985.19	1954	47.77	41	.934	.001	.924	.038	.035 (.032, .037)	.000
	Residual invariance	3098.80	1961	113.61	7	.928	.006	.917	.073	.037 (.034, .039)	.002
41 (*n* = 421)	Configural invariance	Insufficient sample									
42 (*n* = 317)	Configural invariance	Insufficient sample									
43 (*n* = 509)	Configural invariance	Insufficient sample									
44 (*n* = 294)	Configural invariance	Insufficient sample									
45 (*n* = 407)	Configural invariance	Insufficient sample									
46 (*n* = 422)	Configural invariance	Insufficient sample									
47 (*n* = 219)	Configural invariance	Insufficient sample									
48 (*n* = 431)	Configural invariance	Insufficient sample									

Subsample	Model	χ^2	*df*	$\Delta\chi^2$	Δ*df*	CFI	ΔCFI	TLI	SRMR	RMSEA (90% CI)	ΔRMSEA
49 (*n* = 821)	Configural invariance	2792.19	1626	-	-	.914	-	.880	.914	.042 (.039, .044)	-
	Metric invariance	2878.00	1913	85.81	287	.929	.015	.916	.040	.035 (.032, .038)	.007
	Scalar invariance	2925.30	1954	47.3	41	.928	.001	.917	.040	.035 (.032, .037)	.000
	Residual invariance	2944.10	1961	18.8	7	.927	.001	.916	.042	.035 (.032, .038)	.000
50 (*n* = 1220)	Configural invariance	3094.10	1626	-	-	.912	-	.878	.028	.038 (.036, .041)	-
	Metric invariance	3238.04	1913	143.94	287	.921	.009	.907	.037	.034 (.032, .036)	.004
	Scalar invariance	3292.84	1954	54.8	41	.920	.001	.908	.038	.034 (.032, .035)	.000
	Residual invariance	3296.96	1961	4.12	7	.920	.000	.908	.038	.033 (.031, .035)	.001

Table 3.

Explained common variance, hierarchical omega, subscale omega, and relative omega of general factor and subscales.

Factor	ECV	ω*S*	ω*H*	Relative ω
General	.721	.934	.894	.957
Challenge	.265	.778	.166	.213
Commitment	.195	.791	.078	.099
Emotional Control	.445	.631	.199	.315
Life Control	.188	.739	.126	.170
Confidence in Abilities	.238	.752	.160	.213
Interpersonal Confidence	.409	.726	.278	.383

Note. ECV = Explained Common Variance, ω*S* = Omega Subscale, ω*H* = Omega Hierarchical
